# Supplementary material for: A synergistic mindsets intervention protects adolescents from stress
Source: Nature. 2022 Jul 6;607(7919):512–20. doi: 10.1038/s41586-022-04907-7 (PMC9258473; doi:10.1038/s41586-022-04907-7)
Supplement: Supplementary file 1 — This file contains: Supplementary Table 1, an open science disclosure of study registration, materials, data, and syntax; an explanation of how the pre-registered analysis plan was followed (for Studies 1, 2, and 4) and how researcher degrees of freedom were constrained (for Studies 3, 5, and 6; Supplementary Tables 2–4); disclosure of the covariates included in each study’s BCF analyses (Supplementary Table 5); items used to measure stress mindset and fixed mindset at baseline; evidence of balanced propensity scores across pre-random-assignment covariates (Supplementary Figs. 1 and 2); a detailed description of the Bayesian Causal Forest (BCF) analysis method (Supplementary Fig. 3); plot of the minute-by-minute targeted smoothing for Study 4’s analyses (Supplementary Fig. 4) and frequentist results plotted across studies (Supplementary Fig. 5). [file 41586_2022_4907_MOESM1_ESM.pdf]

nature portfolio

https://doi.org/10.1038/s41586-022-04907-7

Supplementary information

A synergistic mindsets intervention protects adolescents from stress

In the format provided by the authors and unedited

Supplemental Information for:
A Synergistic Mindsets Intervention Protects Adolescents from Stress

Authors:
David S. Yeager[1]*, Christopher J. Bryan[1*], James J. Gross[2], Jared Murray[1], Danielle Krettek[3], Pedro Santos[1], Hannah Graveling[4], Meghann Johnson[1], Jeremy P. Jamieson[4*]

Affiliations:
[1] University of Texas at Austin, Austin, TX, USA
[2] Stanford University, Stanford, CA, USA
[3] Google Empathy Lab, Mountain View, CA, USA
[4] University of Rochester, Rochester, NY, USA

Overview

This online supplement contains the following information:

- Table S1: An open science disclosure of study registration, materials, data, and syntax.
- An explanation of how the pre-registered analysis plan was followed (for Studies 1, 2, and 4) and how researcher degrees of freedom were constrained (for Studies 3, 5, and 6; Tables S2, S3, and S4).
- Disclosure of the covariates included in each study's BCF analyses (Table S5)
- Items used to measure stress mindset and fixed mindset at baseline.
- Evidence of balanced propensity scores across pre-random-assignment covariates (Figures S1 and S2).
- A detailed description of the Bayesian Causal Forest (BCF) analysis method (Figure S3).
- Plot of the minute-by-minute targeted smoothing for Study 4's analyses (Figure S4).
- Frequentist results plotted across studies (Figure S5)

Study	Registration	Materials	Data	Syntax
Study 1	https://osf.io/tgysd*	https://osf.io/6bnyr/	https://osf.io/txrp9/	https://osf.io/rj9dq/
Study 2	https://osf.io/hb6vs*	https://osf.io/fnt9w/	https://osf.io/rx9be/	https://osf.io/f7ms8/
Study 3	https://osf.io/x4a63#	https://osf.io/tdmxb/	https://osf.io/vecwd/; https://osf.io/yh5xw/	https://osf.io/nehs2/
Study 4	https://osf.io/fkgru*	https://osf.io/epa3q/	https://osf.io/75xbs/; https://osf.io/32ecm/	https://osf.io/r5es6/
Study 5	https://osf.io/9pfha#	https://osf.io/s6g7b/	https://osf.io/xe9cj/; https://osf.io/yma8k/; https://osf.io/7ag5y/	https://osf.io/ztyb9/
Study 6	https://osf.io/mkqgf@	https://osf.io/w4r8j/	https://osf.io/d7bvk/	https://osf.io/6dfcp/

Table S1. Open Science Disclosures. * Indicates pre-analysis plan registered prior to accessing data; # Indicates study was registered after analyzing the data, and the analysis plan was based in part on this paper: https://pubmed.ncbi.nlm.nih.gov/27324267/ and a pre-registered replication of that paper: https://osf.io/7my86; @ Indicates study was registered after analyzing the data, and the analysis plan was based on this paper: https://journals.sagepub.com/doi/abs/10.1177/2167702614548317 [1]

Explanation of Analysis Plans

Study 1. Study 1's pre-analysis plan (osf.io/tgysd) stated that we would test four hypotheses, quoted below. Under each, we summarize the results and any deviations from the plan.

H1: The mindset intervention will lead to stronger stress-can-be-enhancing mindsets at immediate post-test.

This analysis was conducted as planned and the hypothesis was supported, as reported in the manuscript.

H2: The mindset intervention will increase positive stress appraisals to a hypothetical challenge in the self-identified most-stressful class (primary outcome).

This analysis was conducted as planned and the hypothesis was supported, as reported in the manuscript. This is called the "response appraisals" in the manuscript.

H3: The mindset intervention will lead to positive changes in secondary outcomes: approach motivation, avoidance motivation, negative stress appraisals, and reappraisal tendencies (secondary outcomes).

The analysis of a composite of the secondary outcomes (e.g., threat/challenge appraisals of the task and feeling energized by the task) was conducted as planned and the hypothesis was supported, as reported in the manuscript. This is called the "event appraisals" in the manuscript.

Additional analyses of approach motivation also yielded the expected effects. For instance, the intervention increased the proportion of students saying they would choose a hard (vs. easy) math assignment during the Fall of 2020, which was mostly online schooling during the pandemic, from 28% in the control condition to 33% in the synergistic mindsets condition, $t = 2.638$, $p = .008$. Because the focus of Study 1 in the paper was on appraisals, however, then we did not include this secondary analysis in the paper.

H4: If there are positive effects on secondary outcomes, they will be stronger among people who report baseline fixed mindset and stress-is-debilitating mindsets. (In past research we have not found moderation of effects on positive stress appraisals by baseline beliefs, so we do not hypothesize that we will find them here).

Although both fixed mindsets and stress-is-debilitating mindsets negatively predicted the primary and secondary outcomes, the intervention effect did not differ by baseline mindsets. However, the focus of Study 1 in the paper was on appraisals, where we did not predict or find moderation by prior mindsets.

Study 2. Study 2's pre-registered analysis plan (osf.io/hb6vs) stated this hypothesis:

H1. We want to know if a "stress-is-enhancing" mindset intervention changes students' responses to a challenging quiz in the first week of psychology class.

The plan listed two primary outcomes. One was negative appraisals of stress responses (e.g. " "I felt like my body's stress responses hurt my performance on today's benchmark"). We supported our hypothesis that the intervention would reduce these appraisals, and this is presented in the manuscript.

Next, the plan listed appraisals of demand ("the quiz was very demanding") and resources ("I had the resources to perform well") as outcomes. The intervention effect on reduced demand appraisals was .14 scale points, $t = 1.897$, $p = .058$. The effect on resource appraisals was null: .06 scale points, $t = 0.793$, $p = .428$. The effect on their ratio was also null.

The plan stated that we would conduct a correlational analysis linking self-reports of synergistic mindsets to the appraisal items, and this analysis appears in the manuscript in Extended Data Table 1.
The plan stated that although we would base all of our conclusions on the appraisal effects, a secondary analysis would explore scores on the benchmark quiz. This analysis will appear in a separate manuscript focused on performance effects of the synergistic mindsets intervention.

Study 3. This study was registered after collecting and analyzing the data (osf.io/x4a63). The study was not pre-registered prior to data collection because (a) this was one of the first studies testing this novel intervention (three were launched simultaneously in the Fall of 2019) and we did not yet know whether the manipulation would change the manipulation check or if data collection would be feasible, and (b) the study procedures, focal DVs, and analysis methods were established by previous published research (Yeager, Lee, & Jamieson, 2016). Note that Study 3 was later subjected to a pre-registered replication: https://osf.io/fkgru. Below, we list different purposes for study pre-registration and explain how this study addressed each.

**Study registration component**	**Study 3's approach to reducing researcher degrees of freedom**
Permanent public record of the study's design, measures, and sample	The study is registered here: https://osf.io/x4a63
Disclose all manipulations	This study included only one manipulation: https://osf.io/x4a63
Study procedures	The study procedures followed the methods in a previously published paper[2].
Data processing rules	Cardiovascular data were processed blind to condition information using rules from a previous publication[2]. Following other pre-registered studies in this paper (https://osf.io/tgysd; https://osf.io/hb6vs), all analyses were "intent-to-treat," which means all participants were included if they had data on the outcome and intervention condition. Thus, the data exclusion rule was not a degree of freedom that was exercised here.
Sample size and stopping rule	The stopping rule was influenced by the study's funder. As stated in the paper, we sought to collect as much data as possible in the Fall 2019 semester, to report results to our study funder by the end of the 2019 calendar year. No data were analyzed until all data had been collected. Thus, the data collection stopping rule was not a degree of freedom that was exercised here.
Covariates	See Table S5
Primary statistical approach (Bayesian Causal Forest)	Study 3 follows the same BCF statistical model outlined in pre-registered Study 1 (https://osf.io/tgysd) and Study 4 (https://osf.io/fkgru). One degree of freedom we could exercise was the particular epoch to focus on. Although we suspected that the speech epoch might show the largest effects, as in Yeager et al. (2016), *Psych Science*, without a pre-analysis plan we decided to analyze the data from all epochs by modeling them flexibly using the targeted-smooth BCF model. This approach gave each epoch the same chance of showing a treatment effect, while also applying a conservative smoothing prior to avoid over-fitting.
Potential moderators	We followed the same moderators as listed in pre-registered Study 1 (https://osf.io/tgysd); Study 2's and 4's pre-registrations did not list moderators. Thus, the inclusion of moderators was not a degree of freedom that was exercised here.
Outcome variables	The outcome variables of TPR, SV, and PEP (during recovery epoch) followed the methods in a previously published paper[2], and were replicated in Study 4 using the same methods and analysis choices. Thus, the choice of the outcome variable was not a degree of freedom that was exercised here.

Table S2. Disclosure of Researcher Degrees of Freedom for Study 3

Study 4. Study 4's pre-analysis plan (https://osf.io/fkgru) listed two hypotheses.

H1: The synergistic mindset intervention condition will show lower threat-type physiological responding (total peripheral resistance, or TPR) relative to the neutral control condition during the stressful speech epoch of the trier social stress test.

This hypothesis was supported, as reported in the manuscript. The analyses followed the pre-registered plan, which called for a new, multi-arm tsBCF analysis of TPR reactivity during the speech epoch. The paper reports the ATE and the 10th to 90th %ile interval for the ATE and the posterior probability that the ATE is different from zero.

H2: Neither the stress mindset-only condition, nor the growth mindset-only condition, will show lower physiological responding (total peripheral resistance, or TPR) relative to the neutral control condition during the stressful speech epoch of the trier social stress test.

This hypothesis was supported, as reported in the manuscript. The analyses followed the pre-registered plan, which called for a multi-arm tsBCF analysis of TPR reactivity during the speech epoch. We did not find meaningfully large or reliable effects for either of the single-mindset conditions, relative to the neutral condition.

Secondary analyses. The plan stated that we would examine Cardiac Output (CO) as a secondary outcome, which indicates stronger challenge-type stress responses. When we conducted this analysis, all hypotheses were supported. However, as mentioned in the paper, the presence of a treatment effect on SNS reactivity (PEP) complicates the use of CO. Therefore, as in the Yeager, Lee, & Jamieson (2016) paper, we used SV rather than CO as a secondary outcome. Hypotheses 1 and 2 were supported when examining SV, as shown in the manuscript.

Study 5. Study 5 was registered after collecting and analyzing the data (https://osf.io/9pfha). In this study, as in our previous studies, including the study design that the present study was modeled on (https://osf.io/7my86), our hypothesis was that the synergistic mindsets intervention would reduce threat-type daily stress responses.

The present study was not pre-registered due to our uncertainty about whether the study design would be feasible with our school partner, which faced many socioeconomic challenges, and how analyses should be conducted to best test our hypotheses given the realities of this sample. In particular, we suspected that the self-reports would operate similarly to our previous studies, but the measures were new because they had to be abbreviated for this study context. In addition, although cortisol is an established indicator of threat responses (see, e.g., Yeager, Lee, & Jamieson, 2016), we did not have clear hypotheses about how to model those potential treatment effects. Because of this uncertainty, rather than pre-register a long decision tree of alternative analysis choices, we decided to (a) register the study and its final design after it had been conducted; (b) constrain our degrees of freedom as much as possible by basing the study design and analysis plan on previously-published studies and analysis plans; (c) be transparent about degrees of freedom that we exercised; and (d) make the data available for further scrutiny if needed. For additional detail, see https://osf.io/9pfha. Below we disclose the degrees of freedom we exercised and our attempts to constrain them.

Study registration component	Study 5's approach to reducing researcher degrees of freedom
Permanent public record of the study's design, measures, and sample	The study is registered here: https://osf.io/9pfha
Disclose all manipulations	This study included only one manipulation: https://osf.io/9pfha
Study procedures	The study procedures were modeled on the Texas Longitudinal Study of Adolescent Stress Resilience (TLSASR), which was pre-registered here: https://osf.io/7my86, and on Study 2 in a previously published paper[2]. The similarities to the TLSASR include the double-blind, student-level randomization of the intervention, measurement of daily intensity of social-evaluative stressors, threat-type self-reports, and salivary cortisol, and all procedures for participant recruitment, consent, and data collection. Differences from the TLSASR include collection of three samples per day (rather than one), an abbreviation of the measure of intensity of social-evaluative stressors, and an abbreviation of the primary self-reported outcome. For space reasons, in the present study we used a single measure of negative self-regard, rather than a composite of threat appraisals and coping outcomes. In summary, the study design was closely modeled on a similar pre-registered study and the adaptations came from an attempt to have more precise estimates and adapt to the realities of working with the school partner.
Data processing rules	All data were processed blind to condition information using rules from a previous publication[2]. We followed the pre-registration for the TLSASR (https://osf.io/7my86) in by conducting analyses with "intent-to-treat" sample, which means all participants were included if they had data on the outcome and intervention condition. Thus, the data exclusion rule was not a degree of freedom that was exercised here.
Sample size and stopping rule	We sought to recruit as many as possible before the end of October in the fall of 2019, because the study was focused on normative stressors at the start of a new school year, because daily diary data collection could not happen during or after the Thanksgiving break in the U.S. (which is in late November), and because we were required to report results to our funder by the end of the 2019 calendar year. The number of students recruited each week was constrained by the research team's capacity to support twice-daily diary surveys and thrice-daily saliva samples in a school environment. The ultimate sample size was determined by the total number of students who could be recruited from the school in the fall semester of 2019 given these constraints. No data were analyzed until all data had been collected. Thus, the data collection stopping rule was not a degree of freedom that was exercised here.
Covariates	See Table S5
Primary statistical approach (Bayesian Causal Forest)	Study 5's focus on the interaction between intensity of daily negative social-evaluative stressors and condition when predicting daily threat-type responses was spelled out in the TLSASR pre-registration (https://osf.io/7my86). Study 5 follows the same BCF statistical model outlined in pre-registered Study 1 (https://osf.io/tgysd) and Study 4 (https://osf.io/fkgru) and mentioned in the TLSASR pre-registration (https://osf.io/7my86; note that the tsBCF method had not been developed at that point). Thus, choice of a statistical model was not a degree of freedom that was exercised here.
Potential moderators	We followed the same moderators as listed in outlined in pre-registered Study 1 (https://osf.io/tgysd). Thus, the inclusion of moderators was not a degree of freedom that was exercised here.
Outcome variables	The outcome variables of threat-type self-reports and cortisol were listed in the TLSASR pre-registration that we based the present study on (https://osf.io/7my86) and in a previously published paper[2]. Thus, the choice of the outcome variables was not a degree of freedom that was exercised here.

Table S3. Disclosure of Researcher Degrees of Freedom for Study 5

Study 6. Study 6 was registered after collecting and analyzing the data (https://osf.io/mkqgf). This study took advantage of the unplanned stressor of the COVID-19 lockdowns in April, 2020, and data collection as a part of a class activity, to evaluate treatment effects on anxiety symptoms. A detailed description of the study's history appears in the registration.

**Study registration component**	**Study 6's approach to reducing researcher degrees of freedom**
Public record of the study's design, measures, and sample	The study is registered here: https://osf.io/mkqgf
Disclose all manipulations	This study included only one manipulation: https://osf.io/mkqgf
Study procedures	The study's random assignment to condition and intervention delivery methods followed Study 2's, with the exception that the post-quiz appraisals were not measured here as in Study 2.
Data processing rules	The anxiety symptom data were processed blind to condition assignment. Following other pre-registered studies in this paper (https://osf.io/tgysd; https://osf.io/hb6vs), all analyses were "intent-to-treat," which means all participants were included if they had data on the outcome and intervention condition. Thus, the data exclusion rule was not a degree of freedom that was exercised here.
Sample size and stopping rule	This study took advantage of an unplanned opportunity that was afforded by the COVID-19 lockdowns in April 2020; participants could not be enrolled after the initial randomization in January, 2020.
Covariates	See Table S5
Primary statistical approach (Bayesian Causal Forest)	The emphasis on reducing internalizing symptoms among those who were higher in baseline values of the mindset targeted by the intervention was based on a previous set of pilot studies we conducted (https://journals.sagepub.com/doi/abs/10.1177/2167702614548317). Study 6 follows the same BCF statistical model outlined in pre-registered Study 1 (https://osf.io/tgysd) and Study 4 (https://osf.io/fkgru). Thus, choice of a statistical model was not a degree of freedom that was exercised here.
Potential moderators	We followed the same moderators as listed in outlined in pre-registered Study 1 (https://osf.io/tgysd). Thus, the inclusion of moderators was not a degree of freedom that was exercised here.
Outcome variables	This was the only measure of anxiety symptoms assessed as a classroom activity. Other psychological measures were also administered by the instructional team as a part of course activities. Although we would not have predicted treatment effects for these other variables, a lack of pre-registration for the outcome variable justifies the higher uncertainty intervals in our Bayesian analyses relative to conventional frequentist analyses.

Table S4. Disclosure of Researcher Degrees of Freedom for Study 6

Covariates

Here we list the covariates that were included in each study. In every case, if a given covariate was not included in the model, it was not measured in that study. Unless otherwise specified, each covariate was chosen because (a) the variable could be related to the outcome, and (b) the variable could show chance differences at baseline, which could lead to spurious results if the model did not adjust for those chance differences. Our philosophy was to include as many covariates as possible when they were measured, and then allow BCF to decide to use them.

**Baseline covariate**	**Justification**	**Study**					
		1	2	3	4	5	6
Fixed mindset*	Baseline value of the variable targeted by the intervention, suspected to influence the outcomes across studies	■	■	■	■	■	■
Stress mindset*	Baseline value of the variable targeted by the intervention, suspected to influence the outcomes across studies	■	■ +	■	■	■	■
Perceived social stress[3]*	Established baseline measure of internalizing symptoms;	■			■	■	■
Sex*	Girls/women tend to show higher internalizing symptoms	■	■	■	■	■	■
Age	Students at different grade levels could experience different academic loads that contribute to their social-evaluative stress	■	■		■	■	■
Race/Ethnicity	Students from different racial or ethnic groups could experience different kinds or intensities of social-evaluative stressors	■			■		■
Self-esteem[4]	Established measure of psychological well-being; expected to be related to outcomes	■ #		■	■	■	
Test anxiety	Highly test-anxious adolescents could be lower-performing and could be more likely to show negative stress reactivity	■	■		■	■	■
Social class	Lower-SES adolescents could be lower-performing and face environmental stressors that relate to internalizing outcomes and prior mindsets.	■	■				■
Personality (BFI)	Requested by reviewer		■				■
Time of day / Day of the week	There is hourly and weekly variation in cortisol levels					■	

Table S5: Covariates Included in BCF Models Across Studies. Note: Grayed-out boxes indicate inclusion of a given covariate. * The "core" moderators are indicated with an *, and they were included in the model whenever they were measured. All results were identical when only including the "core" variables as covariates and excluding all additional covariates. + Only in Study 2, there was an error in the programming of the baseline stress mindset measure, and so data were incomplete for this measure and not usable for the correlational analyses presented in Extended Data Table 1. # Only for Study 1, a single-item measure of self-efficacy was the pre-registered covariate, rather than the global self-esteem measure, because the scenario was very specifically tied to an academic setting (i.e. doing poorly on a stressful assignment) and we sought to control for any potential chance differences in academic confidence across conditions.

Items Used to Measure Stress Mindset and Fixed Mindset at Baseline

Studies 1, 5, and 6
In Studies 1, 5, and 6, survey space was at a premium. Therefore, short versions of the stress mindset and fixed mindset scales were used. They were:

Fixed mindset
You can learn new things, but you can't really change your basic intelligence.
Your intelligence is something about you that you can't change very much.
You have a certain amount of intelligence, and you really can't do much to change it.

Stress-is-debilitating mindset
The overall effect of stress on my life is negative.
Stress makes it harder for me to learn and grow in school.
Stress makes it harder to get things done and reach my goals.

Studies 3 and 4
In Studies 3 and 4, which were lab studies, there was more time for data collection. Therefore, the more expansive version of the baseline stress mindsets was used. These items were:

Stress-is-debilitating mindset
The effects of stress are bad and I should avoid them.
Stress helps me learn and grow. (r)
Stress makes me sick and weak.
Stress helps me work hard and reach my goals. (r)
Stress stops me from learning and growing.
Stress makes me healthy and strong. (r)
Stress makes it hard to get things done and reach my goals.
The effects of stress are good and I should make use of (r)them.

Perceived Social Stress Scale (PSS)

Participants answered these questions for the PSS, rating each on a scale from 1 = *Never* to 5 = *All the time*.

How often have you felt nervous and stressed?
How often have you felt able to control the irritations in your life?
How often have you been upset because of something that happened unexpectedly?
How often have you felt that things were going your way?
How often have you been angered because of things that were outside your control?
How often have you felt confident about your ability to handle your personal problems?
How often have you felt that you were on top of things?
How often have you felt difficulties were piling up so high that you could not overcome them?
How often have you felt that you were unable to control the important things in your life?
How often have you found that you could not cope with all the things you had to do?

Generalized Anxiety Symptoms Inventory

This is the measure used in Study 6 to measure generalized anxiety symptoms.

	Not at all (1)	Several days over the last 2 weeks (2)	Over half the days (3)	Nearly every day (4)
Feeling nervous, anxious, or on edge (1)	○	○	○	○
Not being able to stop or control worrying (2)	○	○	○	○
Worrying too much about different things (3)	○	○	○	○
Trouble relaxing (4)	○	○	○	○
Being so restless it's hard to sit still (5)	○	○	○	○
Becoming easily annoyed or irritable (6)	○	○	○	○
Feeling afraid that something awful might happen (7)	○	○	○	○

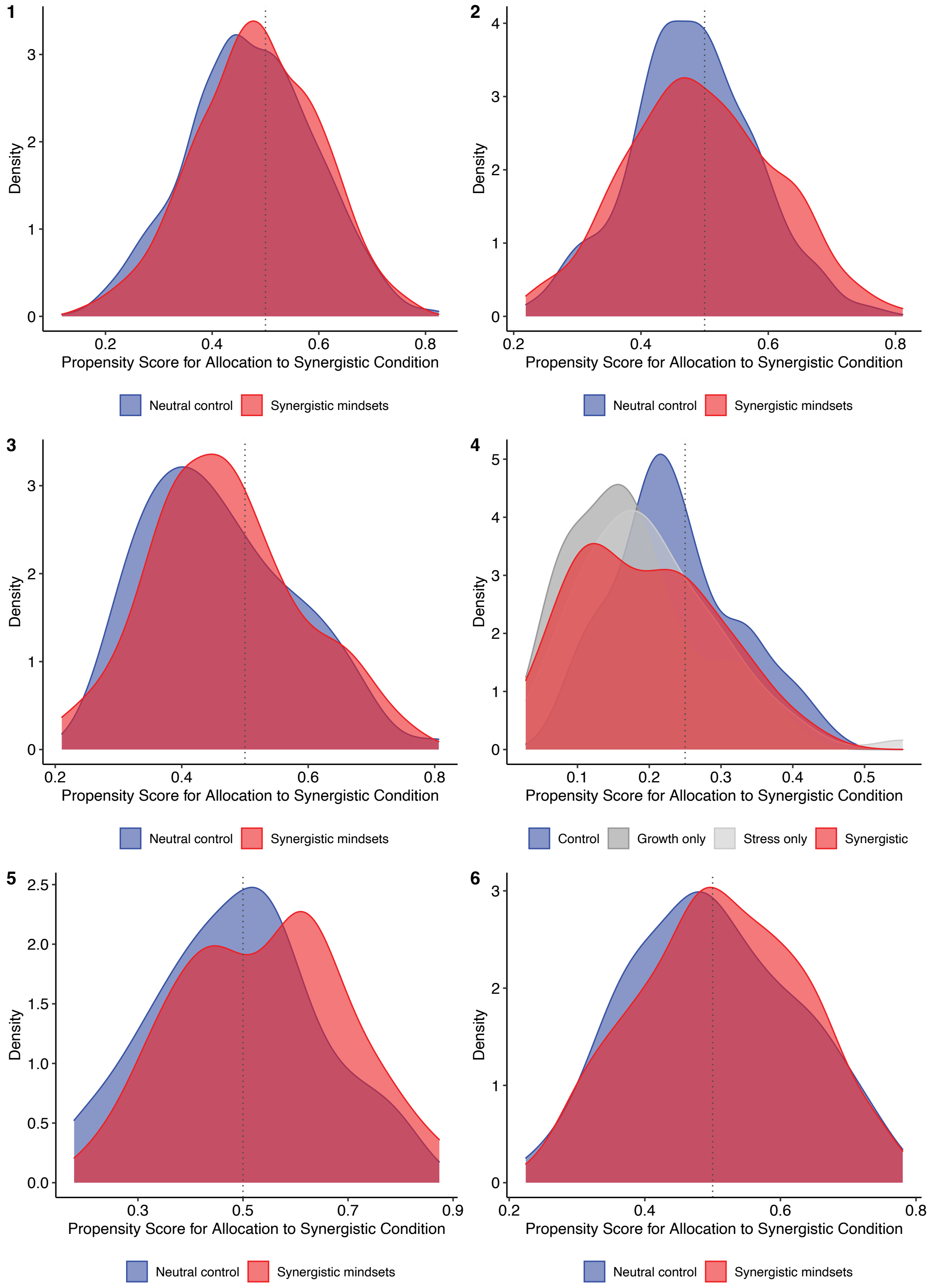

Figure S1. Balanced Propensity Scores Across Conditions, By Baseline Covariates. Note: Numbers are study numbers. Propensity scores estimated using a random forest regression predicting synergistic mindsets condition with the baseline covariates listed in Table S5, separately for each study.

The plots in Figure S1 that the groups were evenly balanced. This is what we expected, of course, because individual-level random assignment was carried out cleanly by the Qualtrics software, and there was little to no attrition in our studies.

The only studies with seemingly meaningful differences across groups were the studies with smaller cell sizes (Studies 4 and 5). This is what would be expected from random noise.

To understand this, we used a random permutation method (in which we randomly shuffled the treatment allocations and then re-ran the propensity score estimation). We found that the magnitudes of the differences in our data were very similar to random noise. For example, below in Figure S2 we present the propensity scores for Study 4, the study with the smallest cell sizes. Panel A shows the propensity score distributions from the observed data. Panel B shows the distributions from randomly shuffled data. The magnitudes of the differences are similar.

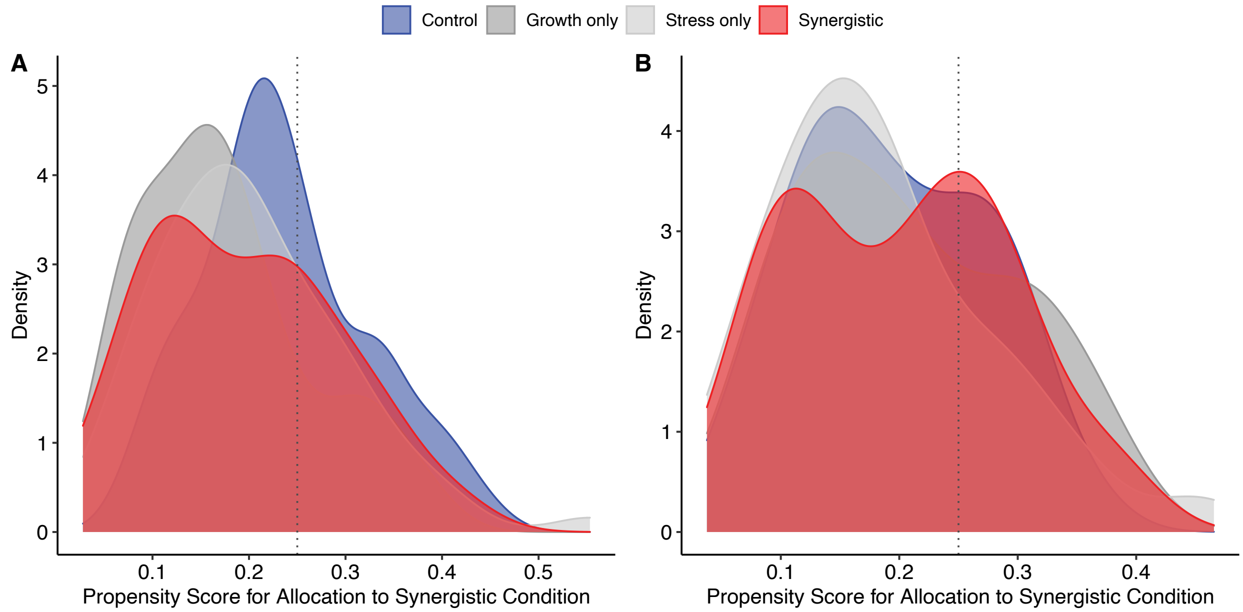

Figure S2. In Study 4, the Modest Imbalances in Baseline Propensity Scores Resemble Random Noise

Further, we note the variables used in the propensity score analyses were also included in the covariate function in the BCF model, which allowed us to correct for any chance imbalances. In summary, the studies' propensity scores were balanced to the extent expected by random variation alone, and our machine-learning model accounted for any observed imbalances.

Descriptive Statistics: Baseline Mindset Values

In each study, a composite of baseline mindset values was constructed by taking the unweighted average of all of the items administered in that study, separately for each construct. The means and standard deviations for the baseline fixed and stress mindsets are depicted in Table S6 below. Note that the two high school studies (1 and 5) showed higher fixed mindset values at baseline. Studies 2 and 6 were conducted at the same university, and Studies 3 and 4 were conducted at the same university. Studies 3 and 4, which were laboratory studies, allowed more time for measurement and therefore used a longer (8-item) measure of stress mindsets.

	Study 1	Study 2	Study 3	Study 4	Study 5	Study 6
Fixed mindset						
M =	3.15	2.52	2.93	2.71	2.99	2.7
SD =	1.12	1.25	1.2	1.1	1.06	1.1
Stress-is-debilitating mindset						
M =	4.27	-	2.9	3.56	3.47	4.1
SD =	1.2	-	0.85	0.84	1.21	1.12

Table S6. Comparisons of baseline values of fixed mindsets and stress mindsets across the six studies. Only in Study 2, there was an error in the programming of the baseline stress mindset measure, and so data were incomplete for this measure and not comparable to the other samples.

Details of the Bayesian Causal Forest (BCF) Analysis

As noted in the paper, BCF has been found, in multiple open competitions and simulation studies, to detect true sources of treatment effect heterogeneity while not lending much credence to noise [5–7]. BCF builds on the popular Bayesian Additive Regression Trees (BART)[8] approach. Both Bayesian regression tree models and BCF in particular have been top performers in empirical evaluations of methods for causal inference [6,7,9,10]. Here we provide more details about how the BCF model was specified and estimated.

In the BCF analysis for studies 1, 2, 5, and 6, the model is specified as

$$y_{ij} = \alpha_i + \beta(x_{ij}) + \tau(w_{ij}) \cdot z_i + \epsilon_{ij},$$

where y_{ij} is outcome j for student i and z_i is a treatment assignment indicator. Here x is a vector of covariates and w is subset of these which are potential treatment effect moderators. These covariates may be measured at the individual level or the observation level. We also allow for individual-level intercept random effects, α_i, to account for varying levels and clustering due to repeated observations in Study 5. In studies without repeated measures, the model omits these terms and the j index. The observation-level error term ϵ_{ij} is assumed to be normally distributed with variance σ^2.

Here β and τ are nonparametric functions which allow for nonlinearities and interactions between covariates in affecting the expected outcome and treatment effects. This model specification is similar to traditional multilevel linear models of heterogeneous treatment effects, but relaxes the strict assumption of linearity and additivity between the covariates and the expected value of the outcome and conditional average treatment effects.

For Study 3, the BART priors on β and τ are replaced by *targeted smooth* BART priors, introduced in [11,12], with time as the smooth variable. These priors are described below. The time series model for Study 3 is specified as

$$y_{it} = \alpha_i + \beta(x_i, t) + \tau(w_i, t) z_i + \epsilon_{it}$$

In Study 4, which uses a tsBCF model with no moderators (to estimate ATEs) and three treatment arms (plus control) we fit a similar model:

$$y_{it} = \alpha_i + \beta(x_i, t) + \tau_{str}(t) z_i^{str} + \tau_{gro}(t) z_i^{gro} + \tau_{syn}(t) z_i^{syn} + \epsilon_{it}$$

Where z_i^{str}, z_i^{gro}, z_i^{syn} are dummy variables indicating assignment to each of the three interventions (stress only, growth only, and synergistic, respectively) and $\tau_{str}(t)$, $\tau_{gro}(t)$, $\tau_{syn}(t)$ are the treatment effects for each condition as a function of time.

Prior specification

To complete our Bayesian model, we must specify prior distributions for the unknown parameters in the equations above. These include the nonparametric functions $\beta(\cdot)$ and $\tau(\cdot)$, the random effects α_i, and the error variance σ^2.

The priors for the functions $\beta(\cdot)$ and $\tau(\cdot)$ in studies 1, 2, 5, and 6 are taken from the Bayesian causal forests model (BCF; Hahn et al., 2020). Under this model, both functions have a sum-of-trees representation, as first defined for Bayesian methods in Chipman, George, and McCulloch (2010). Each tree consists of a set of internal decision nodes which partitions the covariate space, and a set a of terminal nodes, or leaves, corresponding to each element of the partition. The prior for each of $\beta(\cdot)$ and $\tau(\cdot)$ is comprised of three parts: the number of trees, two parameters controlling the depth of each tree, and a prior on the leaf parameters. Use of this sum-of-trees term allows for detection of nonlinearity and interactions between covariates. The prior for the functions $\beta(\cdot)$ and $\tau(\cdot)$ in Study 5 is similar, except it replaces the constant predictions of a BART prior with generic functions of time, assigned Gaussian process prior distributions calibrated as in [11,12]. We use the same Gaussian priors for the functions $\tau_{str}(t),\ \tau_{gro}(t),\ \tau_{syn}(t)$ in Study 4.

A key feature of the BCF model is that the prior for $\tau(\cdot)$, which captures heterogeneity in the intervention effect, is regularized more heavily compared to the control function $\beta(\cdot)$ in order to shrink toward homogeneous effects, i.e. that the intervention effect is constant across all values of the moderators. The prior for $\tau(\cdot)$ uses fewer trees, with each tree being regularized to be shallower (that is, contain fewer partitions). Details are on prior specification are given in [5,8,11,12].

The random effect α_i is given a Gaussian prior with the standard deviation having a prior of a half t-distribution with 3 degrees of freedom, as recommended by Gelman[13]. Finally, the error variance is given an inverse chi-squared prior with 3 degrees of freedom and scale parameter informed by the data.

Posterior Inference and Summarization

To make inferences, we sample from the posterior distribution of the model parameters using a Markov chain Monte Carlo (MCMC) sampling scheme. MCMC sampling for Bayesian sum-of-tree models is described in [5,8,12,14]. Draws from the posterior distribution can then be aggregated to derive posterior distributions for average treatment effects, subgroup average treatment effects, other conditional average treatment effects, or other aggregates or summaries of the model parameters.

We defined subgroups of participants with negative and positive prior mindsets based on their expected outcomes in the absence of our treatment, which is captured by the $\beta(x_{ij})$ term in our model. We treat choosing subgroups as a Bayesian decision problem. We defined a class of possible subgroups based on threshold values of the prior growth mindset and prior stress-can-be-enhancing scales (Figure S3). We chose as a utility function the difference in average outcomes under control between the two groups, so that positive and negative prior mindset groups correspond to those with favorable and unfavorable outcomes in the absence of the intervention. Solving this decision problem entails an exhaustive search for the thresholds that best separate the subgroup average outcomes under control conditions, subject to a minimum sample size of 10.

Finally, to understand the *partial* or *adjusted* effects of some effect moderators—their influence on the estimated treatment effect function when the other moderators are held constant—we use additive spline summaries (or generalized additive models[15]), as described in [16]. The BCF estimate of τ in Studies 3, 5, and 6 was approximately additive (and we found no heterogeneity

in Studies 1-2, as expected). Therefore, to give an interpretable estimate of this conditional intervention effects in Studies 3, 5, and 6, we created an additive summary of the fitted $\tau(\cdot)$ function using splines, and looked at the partial effect of prior negative mindsets, conditional on alternative potential moderators**.** This additive summary closely tracks the fitted τ function itself, so this additive summary is a faithful recapitulation.

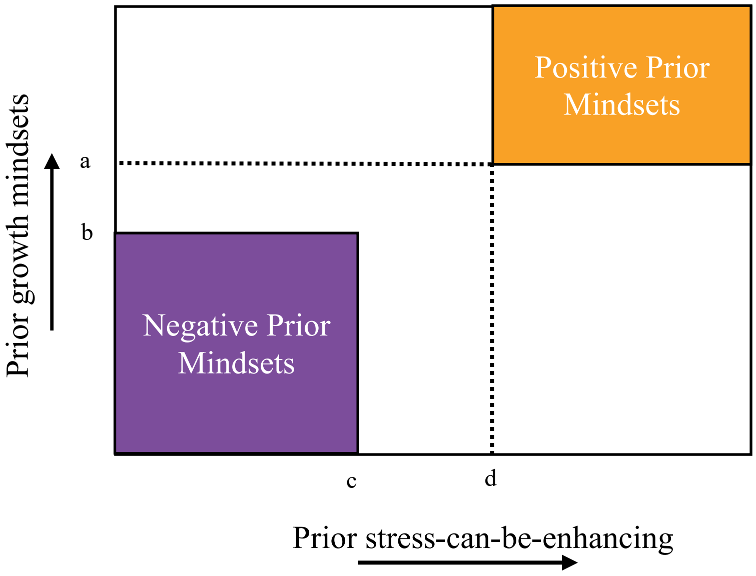

Figure S3. Graphical representation of positive and negative prior mindset subgroups. Note: The positive and negative prior mindsets subgroups are defined by four cutoffs *a,b,c,* and *d*. An adolescent is in the positive prior mindsets group if their scores on the prior growth mindset scale and prior stress-can-be-enhancing scale are above *a* and *d*, respectively, with the negative prior mindsets group defined similarly. The subgroups are defined by searching over the cutoffs to find those which maximize the difference in average outcomes under the control condition between the two subgroups – as estimated by the model – subject to minimum sample sizes in each groups. Note that this process occurs automatically with an unsupervised algorithm, in that researchers do not define the cutpoints *a, b, c,* or *d* by hand. This avoids researcher degrees of freedom concerning the cutpoints chosen.

Minute-by-Minute Targeted Smoothing for Study 4

Study 4's analysis plan focused on treatment effects during the most-stressful epoch during the TSST, the speech epoch. The paper focused on the onset of the stressor, in the first minute of the speech epoch. Here we present the minute-by-minute targeted smoothing for Study 4, which shows that the same conclusions would be obtained by examining all five minutes of the epoch.

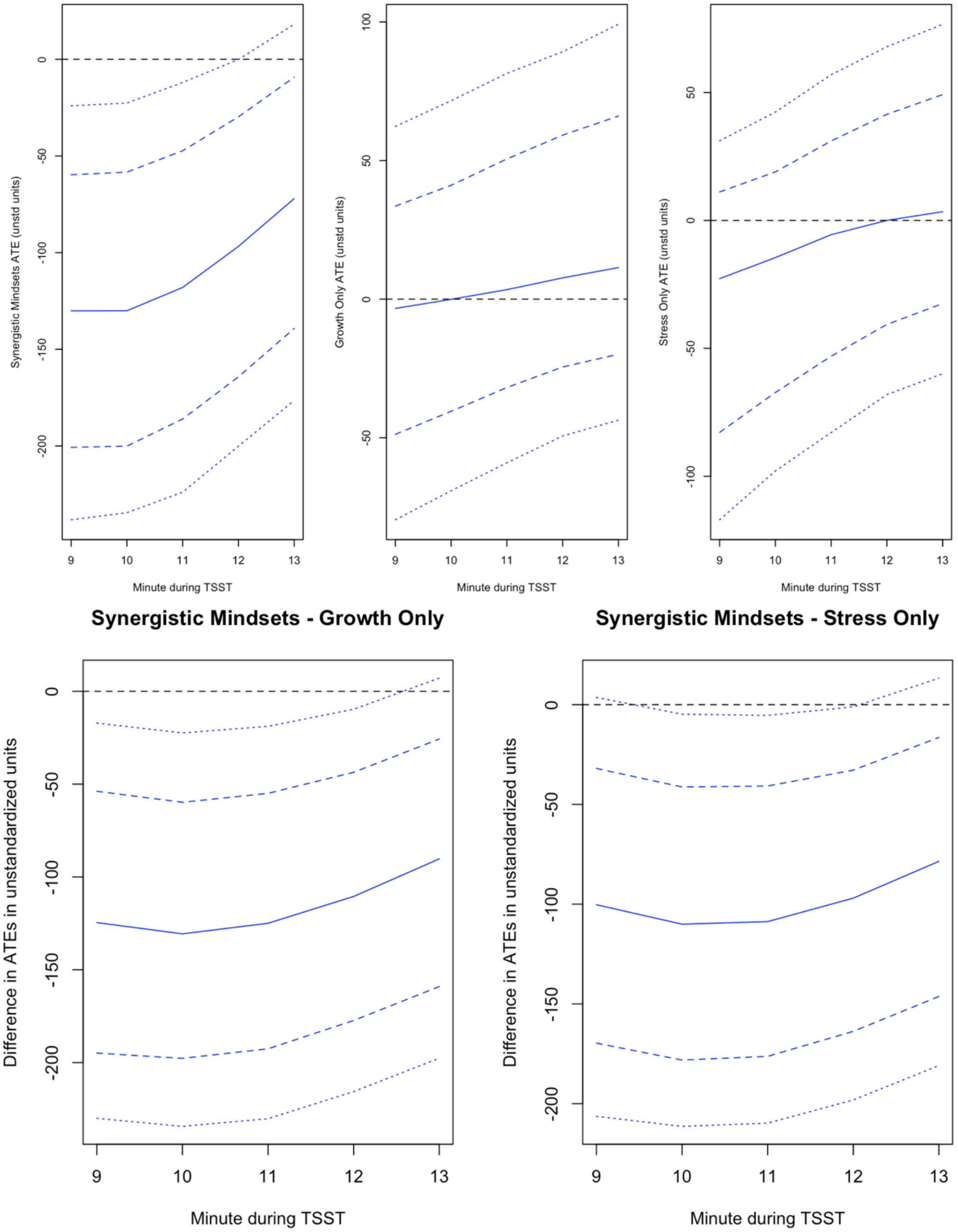

Figure S4. Targeted smoothing within the speech epoch showed larger treatment effects on reduced TPR reactivity for all five minutes of the epoch. Note: The solid lines represent the average treatment effects (ATEs), for the top panel, and the average difference in treatment effects, for the bottom; the dashed lines represent the 10/90th and 2.5/97.5th percentile intervals.

Supplemental Analyses using Conventional Frequentist Methods

When we re-conducted the primary analyses from Studies 1 to 6 using conventional linear models and null-hypothesis significance testing, all conclusions were retained (see individual effect estimates in Extended Data Table 2). Fig. S5 shows that all of the *p*-values were below two conventional cut-points for statistical significance (p <.05 and <.025), and the majority were below the $p < .005$ cut-point (Panel A)[17]. The median effect size was .41 *SD* (Panel B). We caution against dichotomous thinking about the effects being "present" or "absent" [18], because any treatment effect is necessarily heterogeneous. When our BCF analysis method modeled this heterogeneity, it yielded far more uncertainty about the magnitude of the effects, and smaller average effect sizes. We recommend drawing conclusions from the BCF estimates.

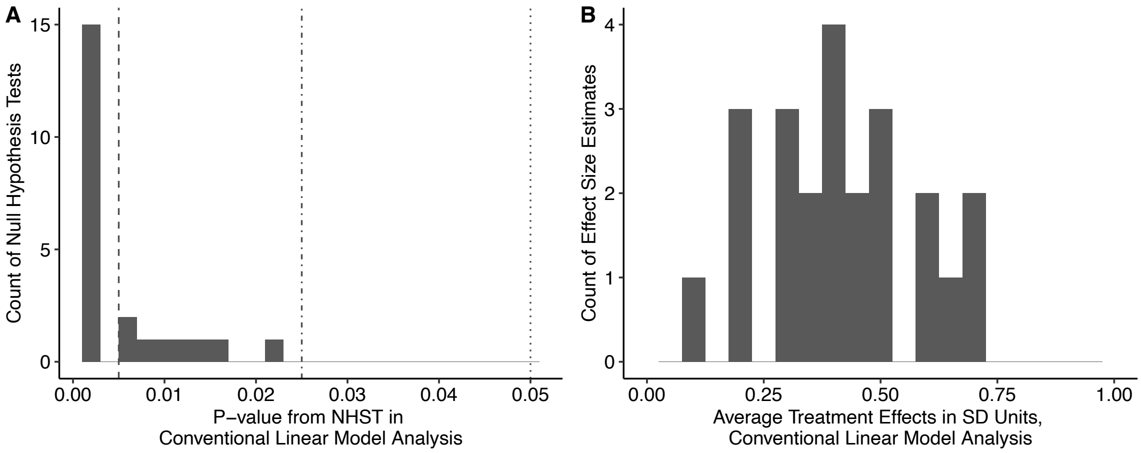

Fig. S5. Conventional linear modeling and null hypothesis significance testing supported the same conclusions concerning the efficacy of the synergistic mindsets intervention across six studies. Note: The raw data appear in Extended Data Table 2. NHST = null hypothesis significance testing. SD = standard deviation.

References

1. Miu, A. S. & Yeager, D. S. Preventing symptoms of depression by teaching adolescents that people can change: Effects of a brief incremental theory of personality intervention at 9-month follow-up. *Clinical Psychological Science* **3**, 726–743 (2015).
2. Yeager, D. S., Lee, H. Y. & Jamieson, J. P. How to improve adolescent stress responses: Insights from integrating implicit theories of personality and biopsychosocial models. *Psychol Sci* **27**, 1078–1091 (2016).
3. Cohen, S., Kamarck, T. & Mermelstein, R. A global measure of perceived stress. *Journal of Health and Social Behavior* **24**, 385–396 (1983).
4. Rosenberg, M. *Society and the adolescent self-image.* (Princeton University Press, 1965).
5. Hahn, P. R., Murray, J. S. & Carvalho, C. M. Bayesian regression tree models for causal inference: regularization, confounding, and heterogeneous effects. *Bayesian Analysis* (2020) doi:10.1214/19-BA1195.
6. McConnell, K. J. & Lindner, S. Estimating treatment effects with machine learning. *Health Services Research* **54**, 1273–1282 (2019).
7. Wendling, T. *et al.* Comparing methods for estimation of heterogeneous treatment effects using observational data from health care databases. *Statistics in medicine* **37**, 3309–3324 (2018).
8. Chipman, H. A., George, E. I. & McCulloch, R. E. BART: Bayesian additive regression trees. *The Annals of Applied Statistics* **4**, 266–298 (2010).
9. Dorie, V., Hill, J., Shalit, U., Scott, M. & Cervone, D. Automated versus do-it-yourself methods for causal inference: Lessons learned from a data analysis competition. *Statist. Sci.* **34**, 43–68 (2019).

10. Hahn, P. R., Dorie, V. & Murray, J. S. Atlantic Causal Inference Conference (ACIC) Data Analysis Challenge 2017. *arXiv preprint arXiv:1905.09515* (2019).

11. Starling, J. E. *et al.* Targeted Smooth Bayesian Causal Forests: An analysis of heterogeneous treatment effects for simultaneous versus interval medical abortion regimens over gestation. *arXiv:1905.09405 [stat]* (2020).

12. Starling, J. E., Murray, J. S., Carvalho, C. M., Bukowski, R. K. & Scott, J. G. Bart with targeted smoothing: An analysis of patient-specific stillbirth risk. *Annals of Applied Statistics* **14**, 28–50 (2020).

13. Gelman, A. Prior distributions for variance parameters in hierarchical models (comment on article by Browne and Draper). *Bayesian Anal.* **1**, 515–534 (2006).

14. Hill, J., Linero, A. & Murray, J. Bayesian Additive Regression Trees: A Review and Look Forward. *Annual Review of Statistics and Its Application* **7**, 251–278 (2020).

15. Hastie, T. & Tibshirani, R. *Generalized additive models*. (Chapman and Hall, 1990).

16. Woody, S., Carvalho, C. M. & Murray, J. S. Model interpretation through lower-dimensional posterior summarization. *arXiv:1905.07103 [stat]* (2020).

17. Benjamin, D. J. *et al.* Redefine statistical significance. *Nature Human Behaviour* **2**, 6–10 (2018).

18. Bryan, C. J., Tipton, E. & Yeager, D. S. Behavioural science is unlikely to change the world without a heterogeneity revolution. *Nat Hum Behav* **5**, 980–989 (2021).
