# Supplementary material for: A synergistic mindsets intervention protects adolescents from stress
Source: Nature. 2022 Jul 6;607(7919):512–20. doi: 10.1038/s41586-022-04907-7 (PMC9258473; doi:10.1038/s41586-022-04907-7)
Supplement: Supplementary file 2 — Reporting Summary [file 41586_2022_4907_MOESM2_ESM.pdf]

nature portfolio

Corresponding author(s): Yeager

Last updated by author(s): Mar 26, 2022

Reporting Summary

Nature Portfolio wishes to improve the reproducibility of the work that we publish. This form provides structure for consistency and transparency in reporting. For further information on Nature Portfolio policies, see our Editorial Policies and the Editorial Policy Checklist.

Statistics

For all statistical analyses, confirm that the following items are present in the figure legend, table legend, main text, or Methods section.

n/a	Confirmed	
☐	☒	The exact sample size (n) for each experimental group/condition, given as a discrete number and unit of measurement
☐	☒	A statement on whether measurements were taken from distinct samples or whether the same sample was measured repeatedly
☐	☒	The statistical test(s) used AND whether they are one- or two-sided<br>*Only common tests should be described solely by name; describe more complex techniques in the Methods section.*
☐	☒	A description of all covariates tested
☐	☒	A description of any assumptions or corrections, such as tests of normality and adjustment for multiple comparisons
☐	☒	A full description of the statistical parameters including central tendency (e.g. means) or other basic estimates (e.g. regression coefficient) AND variation (e.g. standard deviation) or associated estimates of uncertainty (e.g. confidence intervals)
☐	☒	For null hypothesis testing, the test statistic (e.g. F, t, r) with confidence intervals, effect sizes, degrees of freedom and P value noted<br>*Give P values as exact values whenever suitable.*
☐	☒	For Bayesian analysis, information on the choice of priors and Markov chain Monte Carlo settings
☐	☒	For hierarchical and complex designs, identification of the appropriate level for tests and full reporting of outcomes
☐	☒	Estimates of effect sizes (e.g. Cohen's d, Pearson's r), indicating how they were calculated

Our web collection on statistics for biologists contains articles on many of the points above.

Software and code

Policy information about availability of computer code

Data collection	None
Data analysis	The open source software, Multibart 0.3, and syntax for each study, are available at https://osf.io/3zmqc/.

For manuscripts utilizing custom algorithms or software that are central to the research but not yet described in published literature, software must be made available to editors and reviewers. We strongly encourage code deposition in a community repository (e.g. GitHub). See the Nature Portfolio guidelines for submitting code & software for further information.

Data

Policy information about availability of data

All manuscripts must include a data availability statement. This statement should provide the following information, where applicable:

- Accession codes, unique identifiers, or web links for publicly available datasets
- A description of any restrictions on data availability
- For clinical datasets or third party data, please ensure that the statement adheres to our policy

The data are available on osf.io (https://osf.io/3zmqc/).

Field-specific reporting

Please select the one below that is the best fit for your research. If you are not sure, read the appropriate sections before making your selection.

☐ Life sciences ☒ Behavioural & social sciences ☐ Ecological, evolutionary & environmental sciences

For a reference copy of the document with all sections, see nature.com/documents/nr-reporting-summary-flat.pdf

Behavioural & social sciences study design

All studies must disclose on these points even when the disclosure is negative.

Study description

Six between-subjects randomized, controlled intervention experiments.

Research sample

All samples were adolescents in secondary and post-secondary education and were chosen because they were expected to be undergoing social-evaluative stressors that are common to formal educational settings. The samples in each study were not representative of the U.S. population but they were representative, universal samples of their respective schools (i.e. no selection criteria were applied when inviting participants to join the study, except for the medical criteria listed for Studies 3 and 4). Study 1: Participants were from a large, heterogeneous sample of adolescents who were evenly distributed across grades 8 to 12 in 35 U.S. public schools (13 y/o: 16%; 14: 20%; 15: 20%; 16: 21%; 17: 18%; 18: 5%). The schools were sampled from a stratum of large, diverse, suburban and urban public schools in the southeast United States. Forty-nine percent of adolescents identified as male, 49% as female, and 2% as gender non-binary. Participants were racially and ethnically diverse (participants could indicate multiple racial/ ethnic identities so numbers exceed 100%): Black: 20%; Latinx: 39%; White: 68%; Asian: 7%. Participants were also socioeconomically diverse: 40% received free or reduced-price lunch, an indicator of low family income. Study 2: Participants were predominately first-year college students attending a selective public university in the United States that drew from a wide range of socioeconomic status groups: 17 years-old: 3%; 18: 49%; 19: 29%; 20: 11%: 21 or older: 8%. Sixty-four percent identified as female and the rest as male; 39% had mothers who did not have a four-year college degree or higher (an indicator of lower socioeconomic status), and 59% identified as lower class, lower middle class, or middle class (vs. upper middle or upper class). Study 3: Participants were prescreened and excluded for physician-diagnosed hypertension, a cardiac pacemaker, BMI > 30, and medications with cardiac side effects. A total of 166 students were recruited from a university social science subject pool (120 females, 46 males; 76 White/Caucasian, 12 Black/ African-American, 17 Latinx, 65 Asian/Asian-American, 2 Pacific Islander, 4 Mixed Ethnicity, 7 Other; Mage = 19.81, SD = 1.16, range = 18–26; 32% reported their mothers did not have a college degree). Study 4: Participants were from the same university pool as Study 3 and were recruited using the same protocols and exclusion criteria. A total of 200 students provided valid TPR data (163 females, 37 males; 79 White/Caucasian, 22 Black/African-American, 14 Latinx, 79 Asian/Asian-American, 6 Other; Mage = 20.11, SD = 1.77, range = 18–32; 32% reported their mothers did not have a college degree). Study 5: Participants were adolescents from economically-disadvantaged families (99%); 78% were Black/African-American, 5% were White or Asian, and the remaining students were Hispanic/Latino; 36% were in 9th grade; 34% were in 10th grade; 18% were in 11th grade; 12% were in 12th grade. Students attended a high-quality urban charter school which showed a high graduation rate (98%) relative to the urban city school district (68%). The teachers at the school were well-trained and motivated, having earned a national distinction for this charter school. Therefore, the synergistic mindsets intervention was not expected to overcome an absence of objective opportunities to learn, but rather to inspire students to take advantage of the opportunities for upward mobility. Study 6: Data were collected during the Spring semester of 2020. Participants were from the same university as Study 2, and the demographics were nearly identical to Study 2, and the same intervention procedures were followed.

Sampling strategy

All students used convenience sampling methods, with the exception of Study 1, which used a stratified sampling method within the Character Lab Research Network's pool. As noted above, all samples were universal, in that no restrictions were placed on participation in the study (i.e. participants were not screened out for inability to read or speak English, learning disabilities, etc.). In Studies 3-4, participants were prescreened and not invited to participate in the study for physician-diagnosed hypertension, a cardiac pacemaker, BMI > 30, and medications with cardiac side effects. Here is how sample sizes were determined: Study 1: We requested a "fully-powered" sample from CLRN and the exact sample was determined by CLRN. This sample size was planned to have sufficient power to detect a treatment effect in a field experiment of .10 SD or greater, with .10 SD being the minimum effect size that we would interpret as meaningful for a study focused on immediate post-test self-reports. Study 2: All students in an introductory social science course in Fall 2019 were invited to complete the intervention or control materials in return for a small amount of course credit. Sample size was set by the response rate. Study 3: An a priori power analysis was used to determine sample size. Previous stress research that assessed cardiovascular responses in laboratory-based stress induction paradigms produced medium to large effect sizes (e.g., range: d = .59 to d = 1.44 in Yeager et al., 2016, Jamieson et al., 2012, Oveis et al., 2020). Based on a standard medium effect size, at the low end of this range (d = 0.50), with a two-tailed hypothesis, G*Power indicated that 64 participants per condition (i.e., 128 total participants) would be necessary to achieve a target power level of .80 to test for basic effects of the treatment using frequentist methods. In anticipation of potential data loss, we determined a priori that we would oversample by 20%. Data collection was terminated the week after more than 150 participants had been enrolled in the study and provided valid data. Study 4: Study 3 showed an ATE for the synergistic mindsets intervention of approximately .70 SD for TPR reactivity during the first minute of the speech epoch. In this preregistered replication of Study 3, assuming an approximately 25% reduction in effect size for a replication study, then to have an 80% likelihood of reliably detecting an ATE of .50 SD with a one-tailed hypothesis test (because this is a replication study), we calculated that we would need approximately 50 participants per condition. Our stopping rule was to collect data from 200 participants who completed one of the conditions and provided valid TPR data for analysis. Study 5: We aimed for a minimum of 100 participants and 1,000 daily diary responses in this first-ever field experiment evaluating the synergistic mindsets treatment. We sought to recruit as many as possible before the end of October in the fall of 2019, because the study was focused on normative stressors at the start of a new school year, and because daily diary data collection could not happen during or after the Thanksgiving break in the U.S. (which is in late November). The number of students recruited each week was constrained by the research team's capacity to support twice-daily diary surveys and thrice-daily saliva samples in a school environment. The ultimate sample size was determined by the total number of students who could be recruited from the school in

the fall semester of 2019, given these constraints. Study 6: We recruited all students possible from an entire social science class in the spring of 2020, which, we would later learn, was a unique cohort for examining stress during the COVID-19 lockdowns. A minimum of 278 students would be needed to have a greater than 80% chance of detecting a directional effect on anxiety of .3 SD with a conventional linear model analysis, and more students than this participated.

Data collection

In all studies, experimenters, teachers, and anyone else present were kept blind to study hypotheses and condition assignment (which was determined randomly by the survey software). Data collection occurred via web-based surveys in Qualtrics in all studies, except for Studies 3 and 4, which also involved laboratory measures of cardiovascular responding, and Study 5, which involved paper data collection for daily surveys as well as daily saliva sampling. In Study 1, students completed surveys at scheduled times as a part of their participation in CLRN; about half of students were doing remote learning due to COVID-19, and so they completed the surveys at home, and about half completed them in the computer labs. In Study 2, students completed the intervention and the quiz appraisals on their own computers before class (the intervention) or during class (quiz appraisals); because it is a synchronous online class, students were not in the same room as other participating students except in rare circumstances where students watched the course together. In Studies 3 and 4, only the experimenters and the TSST confederates were in the experimental room with the participants; all were blind to condition assignment. In Study 5, the experimenters were present while participants completed the intervention and the daily surveys; Students completed the interventions individually but with peer participants in the room, due to space limitations at the school; Students completed the daily diaries together with the other students who were participating in a given week; students were not allowed to talk about the study with each other during the data collection sessions. In Study 6, the same procedures as Study 2 were used, except students completed the outcome variable, anxiety symptoms, wherever they were quarantining in April of 2020.

Timing

Study 1 was conducted in the Fall of 2020, during school closures due to COVID-19; Studies 2, 3, and 5 were in the Fall of 2019, prior to the pandemic; Study 4 was conducted in the Fall of 2021, between the Delta variant and the Omicron variant outbreaks; Study 6 was conducted in the Spring of 2020 (from Jan to April).

Data exclusions

Data exclusion rules followed the lab's standard operating procedures and the preregistrations (for studies 1, 2, and 4) and were not changed across studies. Participants were included if they had condition information and provided valid data on the relevant outcome and, for models including moderators, the relevant moderators. That is, all data were included provided that the key variables were present to estimate the model. No additional data exclusions were carried out for studies 1, 2, 5, and 6. In the two laboratory studies, Studies 3 and 4, participants were excluded if the cardiovascular sensors were detached (e.g. if the participant made a sudden movement that disconnected the sensor) or if there were obvious statistical artifacts, as described in the methods for Study 3; this led to the exclusion of 4 participants. In Study 4, because the preregistered primary outcome is TPR reactivity during the speech epoch, participants needed to have at least one minute's worth of TPR data and at least one minute's worth of TPR during the speech epoch. Data were collected until we met the stopping rule of 200 participants with useable data; no data were analyzed with respect to condition effects until that was reached. Thus, in Studies 3/4, all decisions about the TPR data were made blind to condition assignment.

Non-participation

We defined adherence as completion of the last page of the intervention. In the studies where participants were closely supervised by researchers (Studies 3, 4, and 5), adherence was high (97% to 99%). In the studies where the intervention was self-administered with no supervision, adherence was lower but still acceptable: 85%, 88% and 82% for Studies 1, 2, and 6, respectively. Because we conducted intent-to-treat analyses, participants were retained in the analytic sample regardless of intervention completion status.

Randomization

Randomization happened via the Qualtrics survey in real time as participants completed the online intervention materials, at the individual level.

Reporting for specific materials, systems and methods

We require information from authors about some types of materials, experimental systems and methods used in many studies. Here, indicate whether each material, system or method listed is relevant to your study. If you are not sure if a list item applies to your research, read the appropriate section before selecting a response.

Materials & experimental systems

n/a	Involved in the study	
☒	☐	Antibodies
☒	☐	Eukaryotic cell lines
☒	☐	Palaeontology and archaeology
☒	☐	Animals and other organisms
☐	☒	Human research participants
☒	☐	Clinical data
☒	☐	Dual use research of concern

Methods

n/a	Involved in the study	
☒	☐	ChIP-seq
☒	☐	Flow cytometry
☒	☐	MRI-based neuroimaging

Human research participants

Policy information about studies involving human research participants

Population characteristics

These are listed in the "research sample" portion of the reporting summary above.

Recruitment

These are listed in the "research sample" and "sampling strategy" portion of the reporting summary above. Note that the study sites are diverse: Study 1: large urban and suburban school districts; Studies 2 and 6: a large, public university; Studies 3 and 4: A private university; Study 5: An urban public charter school serving students experiencing poverty. Nevertheless,

	the sampled schools were not a random sample of potential schools, and further research will be needed to test the generalizability of the intervention, and to further identify the moderating factors (See Bryan, Tipton, & Yeager, 2021, NHB).
Ethics oversight	Approvals for these studies were obtained from the Institutional Review Boards at the University of Rochester or the University of Texas at Austin. In Studies 2 to 6 active consent were obtained either in writing or through the web-based survey; In Study 1, active student assent was obtained via the CLRN standard operating procedures.

Note that full information on the approval of the study protocol must also be provided in the manuscript.
